# A comparison of methods for excluding light from stems to evaluate stem photosynthesis

**DOI:** 10.1002/aps3.11542

**Published:** 2023-09-04

**Authors:** Nadia A. Valverdi, Camilla Acosta, Gabriella R. Dauber, Gregory R. Goldsmith, Eleinis Ávila‐Lovera

**Affiliations:** ^1^ Schmid College of Science and Technology Chapman University Orange California USA; ^2^ Estación Experimental Agropecuaria Catamarca, Instituto Nacional de Tecnología Agropecuaria Catamarca – La Rioja Catamarca Argentina; ^3^ Smithsonian Tropical Research Institute Balboa, Ancón Panama City Panama

**Keywords:** stem CO_2_ exchange, stem light exclusion, stem surface temperature, stem water vapor conductance

## Abstract

**Premise:**

A comparison of methods using different materials to exclude light from stems to prevent stem CO_2_ exchange (i.e., photosynthesis), without affecting stem conductance to water vapor, surface temperature, and relative humidity, was conducted on stems of avocado trees in California.

**Methods and Results:**

The experiment featured three materials: aluminum foil, paper‐based wrap, and mineral‐based paint. We examined stem CO_2_ exchange with and without the light exclusion treatments. We also examined stem surface temperature, relative humidity, and photosynthetic active radiation (PAR) under the cover materials. All materials reduced PAR and stem CO_2_ exchange. However, aluminum foil reduced stem surface temperature and increased relative humidity.

**Conclusions:**

Methods used to study stem CO_2_ exchange through light exclusion have historically relied on methods that may induce experimental artifacts. Among the methods tested here, mineral‐based paint effectively reduced PAR without affecting stem surface temperature and relative humidity around the stem.

## Resumen

**Premisa:**

Una comparación de diferentes métodos utilizando distintos materiales para bloquear la luz de los tallos y así reducir el intercambio de CO_2_ (fotosíntesis) sin afectar la conductancia del tallo al vapor de agua, su temperatura superficial y la humedad relativa fue llevado a cabo en tallos de árboles de aguacate en California.

**Metodología y resultados:**

El experimento se llevó a cabo utilizando tres materiales: papel de aluminio, papel para envoltura y pintura a base de minerales. Se examinó el intercambio de CO_2_ de los tallos con y sin los materiales de bloqueo de la luz. También se examinó la temperatura de la superficie del tallo, la humedad relativa y la radiación fotosintéticamente activa (PAR por sus siglas en inglés) debajo de los materiales usados para bloquear la luz. Todos los materiales redujeron PAR y el intercambio de CO_2_ del tallo. Sin embargo, el papel aluminio redujo también la temperatura de la superficie del tallo y aumento la humedad relativa.

**Conclusiones:**

Los métodos utilizados para estudiar el intercambio de CO_2_ de los tallos con el ambiente a través del bloqueo de la luz han sido métodos que pueden generar alteraciones no deseadas. Entre los métodos evaluados aquí, la pintura de base mineral fue efectiva reduciendo PAR sin alterar la temperatura superficial del tallo ni la humedad relativa alrededor de este.

Over the past three decades, there has been growing interest in understanding the impact of stem photosynthesis on whole plant carbon gain and water transport (Ávila et al., 2014; Liu et al., 2019; Berry et al., 2021; Tomasella et al., 2021). To study this, researchers have used various strategies to block light from reaching the stem surface and reduce stem gas exchange, i.e., photosynthesis. Methods used in previous studies include nets, aluminum foil, or a combination of both (Table 1). The most commonly used method is loosely wrapping aluminum foil around the stems because of its affordability, ease of application (although time consuming), and high efficacy in blocking light (de Roo et al., 2020a). However, aluminum foil may introduce unwanted experimental artifacts by altering stem surface temperature and relative humidity. Given that photosynthesis is a chemical process that can be heavily influenced by environmental factors like temperature, radiation, and relative humidity, and that stem photosynthesis rates are generally lower than those in leaves (Valverdi et al., 2023), the impact of light exclusion methods on these physical factors needs to be taken into consideration.

**Table 1:** Methods used for stem light exclusion in different tree species.

Light exclusion method used	Primary research question	Plant species	References
Aluminum foil	What is the contribution of stem photosynthesis to growth in this plant species?	*Cytisus scoparius*	Bossard and Rejmanek (1992)
Light‐reducing gauze	The importance of stem‐internal carbon re‐fixation.	*Populus tremula* and *Fagus sylvatica*	Wittmann et al. (2001)
Light‐reducing net	Light‐modulation of cortical CO_2_‐refixation.	*Betula pendula*	Wittmann et al. (2005)
Cloth and aluminum foil	Contribution of woody tissue photosynthesis to trunk growth and bud development.	*Prunus ilicifolia*, *Umbellularia californica*, and *Arctostaphylos manzanita*	Saveyn et al. (2010)
Aluminum foil	The role of corticular photosynthesis in wood production in smooth‐barked branches.	*Eucalyptus miniata*	Cernusak and Hutley (2011)
Aluminum foil	The role of branch photosynthesis in tree functioning.	*Rhizophora apiculata*, *Ceriops australis*, and *Avicennia marina*	Schmitz et al. (2012)
Aluminum foil	The role of woody tissue photosynthesis in tree functioning.	*Populus nigra* ‘Monviso’	Bloemen et al. (2013)
Aluminum foil	The role of woody tissue photosynthesis in tree functioning under drought.	*Populus nigra* ‘Monviso’	Bloemen et al. (2016)
Aluminum foil	The role of woody tissue photosynthesis in stem carbon cycling along a gradient of water availability.	*Populus tremula*	de Roo et al. (2020b)
Aluminum foil	Woody tissue photosynthesis under elevated atmospheric CO_2_ concentration.	*Populus tremula*	de Roo et al. (2020a)
Shading net	The role of depletion of non‐structural carbohydrates and xylem vulnerability to embolism.	*Populus nigra*	Tomasella et al. (2021)

Avocado trees have young and mature green stems; this characteristic sets them apart from other fruit trees and may play an essential role in their water and carbon balances through stem photosynthesis (Esteban et al., 2010). In this study, we compared new and existing methods for excluding light from stems using three different materials including aluminum foil, paper wrap, and mineral‐based paint. Our aim was to effectively halt stem gas exchange without significantly altering the surface temperature and relative humidity around the stem or affecting stem conductance to water vapor.

## METHODS AND RESULTS

This experiment was carried out on field‐grown avocado trees of the Gem cultivar (*n* = 5) in a common garden at the South Coast Extension and Research Center (SCERC) of the University of California in Irvine, California, USA. To investigate the effects of different light restriction methods on stem photosynthesis, we selected four sun‐exposed secondary lateral branches on each tree. The branches were at a height midway to the tree canopy and one to two years old to ensure they were photosynthetically active.

**Figure A1:**
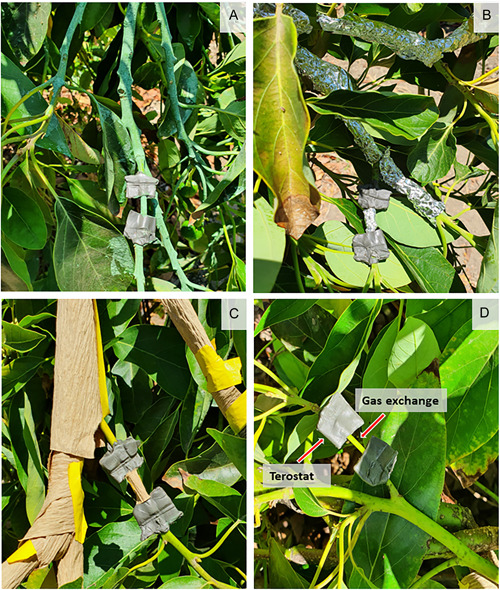
Three light exclusion methods used to reduce stem CO_2_ exchange in avocado stems, including (A) branch covered with mineral‐based paint, (B) aluminum foil–wrapped branch, (C) paper‐wrapped branch, and (D) control branch.

We applied four light restriction methods to the branches: the first branch was coated with three layers of green mineral‐based paint (White Wash Plant Guard; IV Organics, Los Angeles, California, USA); a second branch was covered with aluminum foil (Reynolds Wrap; Alcoa, Pittsburg, Pennsylvania, USA); a third branch was covered using a light brown, breathable paper wrap (model no. 350, 6 m long × 10 cm high; Bond Manufacturing, Antioch, California, USA) secured with adhesive paper tape (Roll Products, St. Marys, Kansas, USA) (Appendix 1, Figure A1); and a fourth branch was left uncovered as a control.

For gas exchange measurements, we used a portion of the branch with a diameter of approximately 0.5–0.8 cm and a length of 3 cm, which could fit in the gas exchange chamber (3 × 3 cm leaf chamber; LI‐COR Biosciences, Lincoln, Nebraska, USA). We performed gas exchange measurements after one and two weeks of covering the branches (i.e., at weeks 2 and 3 of the experiment) to evaluate the effects of light blockage on stem gas exchange over time.

**Figure 1:**
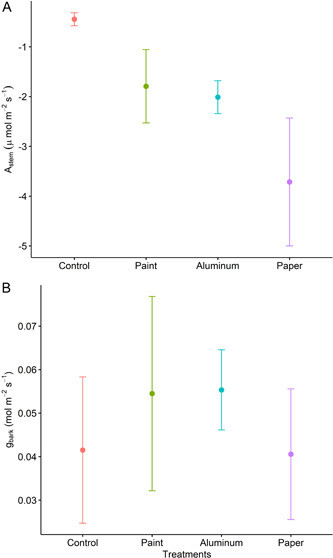
Stem CO_2_ exchange (A) and bark conductance to water vapor (B) for avocado stems covered with mineral‐based paint, aluminum foil, and paper wrap as light exclusion treatments, as well as a control (*n* = 5, points represent mean ± SE).

Gas exchange measurements were taken between 1100 hours and 1400 hours using an infrared gas analyzer (6800, LI‐COR Biosciences) with a 3 × 3 cm leaf chamber in which the gaskets were lined with rubber foam and Terostat adhesive (Teroson; Henkel Corporation, California, USA), allowing for a tight seal around the stem with no significant leaks (Ávila‐Lovera et al., 2017). Measurements were performed at 410 µmol·mol^−1^ of CO_2_, 1500 µmol·m^−2^·s^−1^ of PAR, 400 µmol·s^−1^ of flow rate, 10,000 RPM fan speed, 50% relative humidity, and 25°C temperature. Because the stems did not cover the whole leaf chamber, gas exchange values were recalculated using the stem surface area, which was calculated using the formula of the area of a cylinder without the top and bottom circles (i.e., 2π ∗ r ∗ l, where *r* = the radius of the stem used and l = the length). Results showed that all cover materials (paint, aluminum, and paper) effectively reduced the stem CO_2_ exchange compared to the control (*P* = 0.04) (Figure 1A). The paper wrap demonstrated the greatest absolute reduction in CO_2_ exchange rates relative to the control. On the other hand, there were no significant differences between treatments for bark conductance (*g*_*bark*_), although the paper and aluminum treatments tended to increase *g*_*bark*_ when compared to the control (Figure 1B).

**Figure 2:**
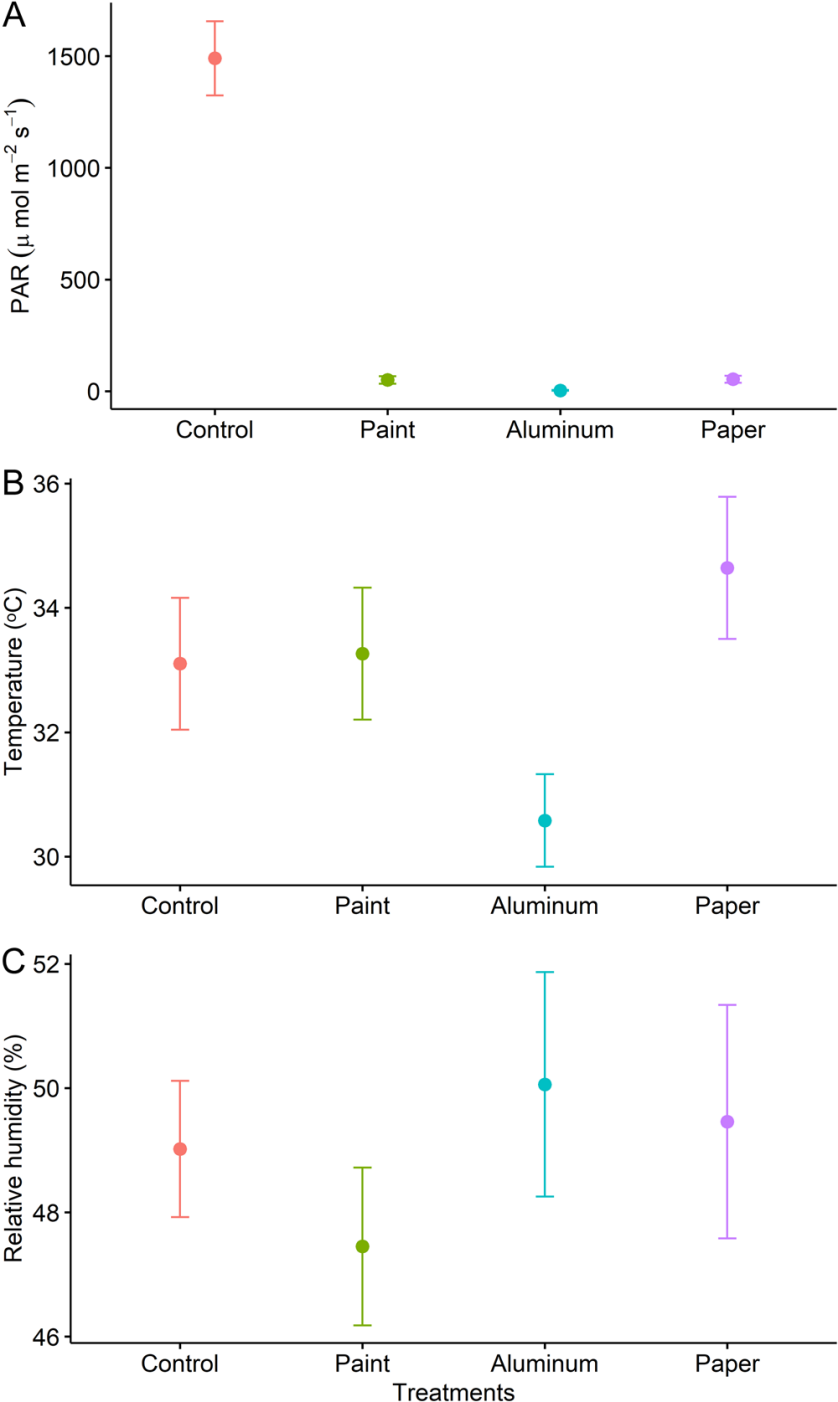
Photosynthetically active radiation (PAR) (A), stem surface temperature (B), and relative humidity (C) for avocado stems covered with mineral‐based paint, aluminum foil, and paper wrap as light exclusion treatments, as well as a control (*n* = 5, points represent mean ± SE).

**Figure A2:**
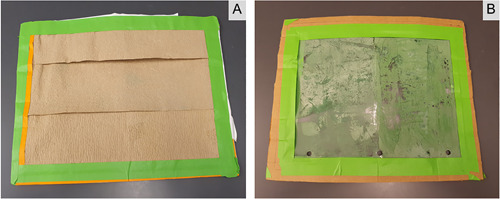
Examples of cardboard frames containing different cover materials to hold above the light meter and measure photosynthetic active radiation (PAR) in the field under a clear sky: (A) paper‐based wrap and (B) transparent PET covered with mineral‐based paint.

To measure stem surface temperature, we used a copper‐constantan thermocouple (Extech model 421509 with Type K Thermocouple; Extech Instruments, Nashua, New Hampshire, USA), while relative humidity was measured with a precision psychrometer (RH390; Extech Instruments). Photosynthetic active radiation (PAR) was measured with a light meter (Li‐250A, LI‐COR Biosciences) at 1200 hours on the same days that gas exchange measurements were made. To measure PAR, we secured each of the different cover materials (paper wrap, aluminum foil, or a transparent polyethylene terephthalate [PET] plastic sheet with three layers of paint) in separate cardboard frames. The frames were held parallel to the ground under a clear sky above the light meter sensor (Appendix 1, Figure A2). Our results showed that all cover materials effectively reduced PAR to nearly zero (*P* < 0.001) (Figure 2A). We found that the covers had a significant effect on stem surface temperature (*P* = 0.02), with aluminum foil having the lowest value. Relative humidity was not significantly affected by the covers (*P* = 0.5). In general, the aluminum foil cover had the lowest stem temperature and the highest relative humidity (Figure 2B, C).

It is challenging to provide an accurate cost comparison of these materials due to fluctuating prices and differences in the amount of branch material to be covered. For the new mineral‐based paint method, we estimate from a different experiment that we used ca. 30 mL of paint per layer per potted tree (2 m tall). The cost of this paint was $32.50 USD for 473 mL (i.e., 1 U.S. pint), making it the most expensive method. However, the paint creates very little waste compared to aluminum foil and paper wrap, which are difficult to adjust due to the different branch sizes and bifurcations. Additionally, applying the paint is relatively fast (e.g., it dries quickly between layers), especially in comparison to the paper. It is worth noting that the paint is water‐soluble, so it will wash off when it becomes wet from rain/irrigation. While the paint is durable, we have previously observed cracking on trees in the field after 4–6 weeks (personal observation).

### Statistical analysis

To test the differences among treatments, we used a linear mixed model with time (data from week 1 and week 2) as a random effect using the package lmerTest in R version 3.4.0 (R Core Team, 2017).

## CONCLUSIONS

In this study, we compared different methods for creating a light exclusion environment to assess stem CO_2_ exchange. In addition to the commonly used aluminum foil (see Table 1), we proposed new materials, such as using paper wrap and mineral‐based paint on avocado trees. All of these cover methods effectively blocked light from reaching the stem and altered stem CO_2_ exchange without significantly affecting bark conductance to water vapor.

However, we observed that the use of aluminum foil resulted in reduced stem surface temperature (Figure 2), leading to mold formation (personal observation). The paper wrap was found to increase stem surface temperature, but we did not observe mold formation using this material. The paper wrap, however, was the most time‐consuming method as it required tape to hold the paper in place, and the material was not very malleable. Overall, we found that the paint cover was the easiest and least time‐consuming method to use, and it also had the least impact on stem temperature and relative humidity while effectively blocking light and reducing stem CO_2_ exchange. This method is especially useful for experiments carried out in dry climates such as Southern and Central California, where the irrigation system does not wet the tree stems.

## AUTHOR CONTRIBUTIONS

N.A.V., C.A., G.R.D., G.R.G. and E.A.L. made substantial contributions to conception and design, acquisition of data, analysis, and interpretation of data; were involved in drafting the manuscript or revising it critically for important intellectual content; and approved the final version of the manuscript. All authors have agreed to be accountable for all aspects of the work in ensuring that questions related to the accuracy or integrity of any part of the work are appropriately investigated and resolved.

## Supporting information


**Appendix S1.** Stem photosynthesis (A_stem_), stomata conductance (gs), air temperature (T), relative humidity (HR), and photosynthetically active radiation (PAR) for three light exclusion treatments (paint, aluminum, paper) and an untreated control on avocado plants (*n* = 5).

## Data Availability

All supporting data are available within the published article as Supporting Information (Appendix S1).

## Appendix 1 Protocol for blocking the light from stems in avocado trees.

**Materials**

- Aluminum foil wrap (Reynolds Wrap; Alcoa, Pittsburg, Pennsylvania, USA)
- Paper wrap (model no. 350, 6 m long × 10 cm high; Bond Manufacturing, Antioch, California, USA)
- Mineral‐based paint (White Wash Plant Guard; IV Organics, Los Angeles, California, USA)
- Paper tape (Roll Products, St. Marys, Kansas, USA)
- Scissors
- Paintbrush
- Water
- Terostat adhesive (Teroson; Henkel Corporation, California, USA)

**Procedure**

1. Select four sun‐exposed branches in the tree.
2. For the first stem, apply two or three layers of mineral‐based paint to the entire stem using a paintbrush, leaving the leaves and petioles uncovered.
3. For the second branch, cover all stem sections with aluminum foil, leaving only the leaves and petioles uncovered.
4. For the third branch, cover as much of the stem as possible with paper wrap, leaving only the leaves and petioles uncovered.
5. Leave the fourth branch uncovered as a control.
6. On all four branches, select a piece of stem between 5 and 8 mm in diameter to measure stem gas exchange and wrap two pieces of Terostat adhesive approximately 3 cm apart to ensure the stem pieces will fit into the gas exchange analyzer chamber.
7. Cut a small piece of aluminum foil or paper wrap, or apply paint to cover the piece of stem between the two Terostat pieces, except for the uncovered control (Figure A1).
8. One to two weeks after covering the branches, measure stem gas exchange as explained in the Methods section.
9. For PAR measurements, make two frames using cardboard or any available material to hold the paper wrap and a polyethylene sheet covered with mineral paint. Place the PAR sensor on a flat surface perpendicular to the floor and place the frame with the light exclusion material above it (Figure A2).
10. For temperature and relative humidity measurements, place the sensor's head under a loose covering material without uncovering the branch, touching the stem surface.

**Note:** Make sure the trees are well irrigated and monitor the covered branches for any signs of stress or damage.
