# Supplementary material for: A comparison of methods for excluding light from stems to evaluate stem photosynthesis
Source: Appl Plant Sci. 2023 Sep 4;11(6):e11542. doi: 10.1002/aps3.11542 (PMC10719881; doi:10.1002/aps3.11542)
Supplement: Supplementary file 1 — Appendix S1. Stem photosynthesis (Astem), stomata conductance (gs), air temperature (T), relative humidity (HR), and photosynthetically active radiation (PAR) for three light exclusion treatments (paint, aluminum, paper) and an untreated control on avocado plants (n = 5). [file APS3-11-e11542-s001.docx]

**Appendix S1**. Stem photosynthesis (A_stem_), stomata conductance (gs), air temperature (T), relative humidity (HR), and photosynthetically active radiation (PAR) for three light exclusion treatments (paint, aluminum, paper) and an untreated control on avocado plants (*n* = 5).

| **Week** | **Tree** | **Treatment** | **A_stem_** | **gs** | **T** | **HR** | **PAR** |
| --- | --- | --- | --- | --- | --- | --- | --- |
|  |  |  | (µmol⋅m^−2^⋅s^−1^) | (mol⋅m^−2^⋅s^−1^) | (°C) | (%) | (µmol⋅m^−2^⋅s^−1^) |
| 1 | 1 | Control | −2.597.813.627 | 0.029462131 | 28.5 | 55.4 | 1643.8 |
| 1 | 2 | Control |  |  | 33.2 | 55.1 | 845.9 |
| 1 | 3 | Control | −0.016820798 | 0 | 29.5 | 56.3 | 1704.9 |
| 1 | 4 | Control | −0.618233368 | 0.006056155 | 30.3 | 57.5 | 1751.2 |
| 1 | 5 | Control | −1.725.417.135 | 0.01456439 | 29.6 | 55.1 | 1504.9 |
| 1 | 1 | Paint | −5.293.171.816 | 0.079650015 | 31 | 51.4 | 18.29 |
| 1 | 2 | Paint |  |  | 34.2 | 53.5 | 34.89 |
| 1 | 3 | Paint | −6.079.997.885 | 0.24644284 | 27.4 | 53.5 | 114.48 |
| 1 | 4 | Paint | −1.025.945.058 | 0.004388542 | 30.5 | 55.7 | 50.71 |
| 1 | 5 | Paint | 1.101.782.228 | 0.049637743 | 30.1 | 56.8 | 32.52 |
| 1 | 1 | Aluminum | −6.941.216.807 | 0.034032897 | 27.1 | 53.5 | 1.3 |
| 1 | 2 | Aluminum | −2.244.876.595 | 0.075538053 | 28.2 | 58.3 | 0.19 |
| 1 | 3 | Aluminum | −1.736.327.327 | 0.00702331 | 27.1 | 63.5 | 3.61 |
| 1 | 4 | Aluminum | −1.309.282.089 | 0.055708175 | 29.2 | 56.4 | 3.38 |
| 1 | 5 | Aluminum | −0.13096404 | 0 | 30 | 61.3 | 8.39 |
| 1 | 1 | Paper | −6.901.694.425 | 0.004140361 | 32 | 50.8 | 25.75 |
| 1 | 2 | Paper | −172.319.227 | 0.026852568 | 31.4 | 55.4 | 19.79 |
| 1 | 3 | Paper | −6.971.029.007 | 0.000812929 | 28.9 | 59.4 | 104.89 |
| 1 | 4 | Paper | −106.868.596 | 0.001772958 | 29.2 | 58.4 | 44.89 |
| 1 | 5 | Paper | −4.138.917.472 | 0.032345799 | 30.6 | 67.2 | 70.7 |
| 2 | 1 | Control | 241.918.142 | 0.039528265 | 39.1 | 40 |  |
| 2 | 2 | Control | −0.40134005 | 0.022178308 | 39.2 | 36.7 |  |
| 2 | 3 | Control | −0.560480958 | 0.107400142 | 37.5 | 42.8 |  |
| 2 | 4 | Control | −1.081.678.211 | 0.051692661 | 29.1 | 47.5 |  |
| 2 | 5 | Control | 0.509501833 | 0 | 35 | 43.8 |  |
| 2 | 1 | Paint | −1.344.928.395 | 0.05835588 | 34.9 | 37.7 |  |
| 2 | 2 | Paint | −1.509.535.778 | 0.020161279 | 39.1 | 37.7 |  |
| 2 | 3 | Paint | −0.966013856 | 0.015671019 | 37.8 | 38.8 |  |
| 2 | 4 | Paint | −1.026.195.251 | 0.000692584 | 37.5 | 46.2 |  |
| 2 | 5 | Paint | −0.285564987 | 0 | 30.1 | 43.2 |  |
| 2 | 1 | Aluminum | 12.326.932 | 0.101660417 | 34.2 | 35.9 |  |
| 2 | 2 | Aluminum |  |  | 31.3 | 36.7 |  |
| 2 | 3 | Aluminum | −2.769.938.916 | 0.060695351 | 29.4 | 45.9 |  |
| 2 | 4 | Aluminum | −2.409.808.147 | 0.008904383 | 34.4 | 44.5 |  |
| 2 | 5 | Aluminum | −1.570.565.296 | 0.067121215 | 34.9 | 44.6 |  |
| 2 | 1 | Paper | −5.154.177.154 | 0.183075553 | 45.9 | 34.8 |  |
| 2 | 2 | Paper |  |  | 37.6 | 41.9 |  |
| 2 | 3 | Paper | −7.831.261.481 | 0.092988403 | 37.4 | 40.6 |  |
| 2 | 4 | Paper | −0.308579457 | 0.00441939 | 35.5 | 45.8 |  |
| 2 | 5 | Paper | −1.335.208.404 | 0 | 37.9 | 40.3 |  |
